# Supplementary material for: Hypoxia Rapidly Induces the Expression of Cardiomyogenic Factors in Human Adipose-Derived Adherent Stromal Cells
Source: J Clin Med. 2019 Aug 15;8(8):1231. doi: 10.3390/jcm8081231 (PMC6723458; doi:10.3390/jcm8081231)
Supplement: Supplementary file 1 [file jcm-08-01231-s001.pdf]

Table S1. Statistical significances between normoxic and hypoxic groups in Fig. 3. It was determined by ANOVA using SPSS, and *p* values < 0.05 were considered significant (red).

	ASCs			ADAS#1			ADAS#2			ADAS#3			ADAS#4		
	N:H6	N:H12	N:H24	N:H6	N:H12	N:H24	N:H6	N:H12	N:H24	N:H6	N:H12	N:H24	N:H6	N:H12	N:H24
HIF1A	0.005	0.001	0.018	0.001	0.024	0.027	0.033	0.600	0.071	0.061	0.002	0.072	0.031	0.687	0.232
HIF2A	0.067	0.094	0.001	0.191	0.019	0.018	0.017	0.054	0.023	0.039	0.008	0.005	0.023	0.043	0.252
PPARG	0.161	0.062	0.036	0.128	0.094	0.104	0.074	0.069	0.036	0.156	0.076	0.085	0.049	0.042	0.178
LPL	0.027	0.023	0.022	0.043	0.030	0.002	0.013	0.005	0.024	0.055	0.049	0.053	0.053	0.034	0.053
FABP4	0.060	0.078	0.096	0.242	0.027	0.036	0.063	0.044	0.064	0.003	0.010	0.017	0.037	0.036	0.037
GATA4	0.040	0.043	0.073	0.073	0.594	0.009	0.018	0.010	0.007	0.243	0.077	0.160	0.065	0.061	0.052
TBX5	0.003	0.022	0.037	0.180	0.021	0.041	0.003	0.012	0.172	0.010	0.015	0.124	0.020	0.030	0.010
NKX2.5	0.037	0.038	0.034	0.063	0.056	0.018	0.044	0.623	0.049	0.002	0.033	0.939	0.181	0.005	0.057
SOX9	0.052	0.015	0.048	0.072	0.062	0.053	0.014	0.006	0.006	0.009	0.020	0.006	0.010	0.030	0.019
ACAN	0.425	0.028	0.019	0.004	0.003	0.002	0.153	0.016	0.004	0.009	0.002	0.008	0.007	0.001	0.000
COL2A1	0.024	0.008	0.028	0.005	0.012	0.057	0.027	0.024	0.000	0.011	0.020	0.074	0.894	0.117	0.074
COL1A1	0.001	0.058	0.035	0.017	0.027	0.030	0.007	0.001	0.009	0.025	0.019	0.010	0.000	0.000	0.000
RUNX2	0.019	0.004	0.642	0.029	0.051	0.014	0.057	0.083	0.073	0.070	0.034	0.209	0.052	0.057	0.022
OCN	0.019	0.044	0.047	0.013	0.010	0.029	0.015	0.019	0.018	0.079	0.115	0.076	0.025	0.040	0.038
ALPL	0.906	0.282	0.087	0.750	0.167	0.019	0.017	0.009	0.008	0.047	0.003	0.006	0.004	0.002	0.002

Table S2. Statistical significances between normoxic and hypoxic groups in Fig. 4. It was determined by ANOVA using SPSS, and p values < 0.05 were considered significant (red).

	ASCs			ADAS#1			ADAS#2			ADAS#3			ADAS#4		
	N:H6	N:H12	N:H24	N:H6	N:H12	N:H24	N:H6	N:H12	N:H24	N:H6	N:H12	N:H24	N:H6	N:H12	N:H24
GATA-4	0.025	0.009	0.004	0.004	0.018	0.029	0.003	0.004	0.028	0.000	0.001	0.016	0.001	0.002	0.019
TBX5	0.013	0.005	0.021	0.003	0.001	0.037	0.011	0.006	0.012	0.042	0.001	0.002	0.027	0.019	0.001
TNKX2.5	0.002	0.002	0.052	0.207	0.000	0.000	0.001	0.057	0.001	0.039	0.000	0.000	0.000	0.000	0.001
Troponin T	0.166	0.005	0.310	0.007	0.056	0.005	0.001	0.001	0.005	0.000	0.004	0.000	0.000	0.072	0.000
Myo D	0.025	0.042	0.008	0.007	0.002	0.026	0.000	0.000	0.103	0.013	0.007	0.005	0.062	0.020	0.037
Myosin HC	0.055	0.039	0.262	0.016	0.001	0.002	0.003	0.001	0.066	0.008	0.000	0.001	0.004	0.004	0.001
Caveolin-1	0.348	0.023	0.024	0.071	0.016	0.012	0.034	0.002	0.001	0.000	0.000	0.022	0.002	0.000	0.008

Table S3. Investigated cytokines, chemokines, and acute phase proteins in CM of ASCs and ADASs under normoxic or hypoxic stress using antibody array.

**Adiponectin/Acrp30**	**IFN-gamma**	**CCL2/MCP-1**
Angiogenin	IGFBP-2	CCL7/MCP-3
Angiopoietin-1	IGFBP-3	M-CSF
Angiopoietin-2	IL-1 alpha/IL-1F1	MIF
Apolipoprotein A1	IL-1 beta/IL-1F2	CXCL9/MIG
BAFF/BLyS/TNFSF13B	IL-1ra/IL-1F3	CCL3/CCL4 MIP-1 alpha/beta
BDNF	IL-2	CCL20/MIP-3 alpha
CD14	IL-3	CCL19/MIP-3 beta
CD30	IL-4	MMP-9
CD31/PECAM-1	IL-5	Myeloperoxidase
CD40 Ligand/TNFSF5	IL-6	Osteopontin (OPN)
Chitinase 3-like	IL-8	PDGF-AA
Complement Component C5/C5a	IL-10	PDGF-AB/BB
Complement Factor D	IL-11	Pentraxin 3/TSF-14
C-Reactive Protein/CRP	IL-12 p70	CXCL4/PF4
Cripto-1	IL-13	RAGE
Cystatin C	IL-15	CCL5/RANTES
Dkk-1	IL-16	RBP4
DPPIV/CD26	IL-17A	Relaxin-2
EGF	IL-18 BPa	Resistin
CXCL5/ENA-78	IL-19	CXCL12/SDF-1 alpha
Endoglin/CD105	IL-22	Serpin E1/PAI-1
EMMPRIN	IL-23	SHBG
Fas Ligand	IL-24	ST2/IL1 R4
FGF basic	IL-27	CCL17/TARC
KGF/FGF-7	IL-31	TFF3
FGF-19	IL-32 alpha/beta/gamma	TfR
Flt-3 Ligand	IL-33	TGF-alpha
G-CSF	IL-34	Thrombospondin-1
GDF-15	CXCL10/IP-10	TIM-1
GM-CSF	CXCL11/I-TAC	TNF-alpha
CXCL1/GRO alpha	Kallikrein 3/PSA	uPAR
Growth Hormone (GH)	Leptin	VCAM-1
HGF	LIF	VEGF
ICAM-1/CD54	Lipocalin-2/NGAL	Vitamin D BP

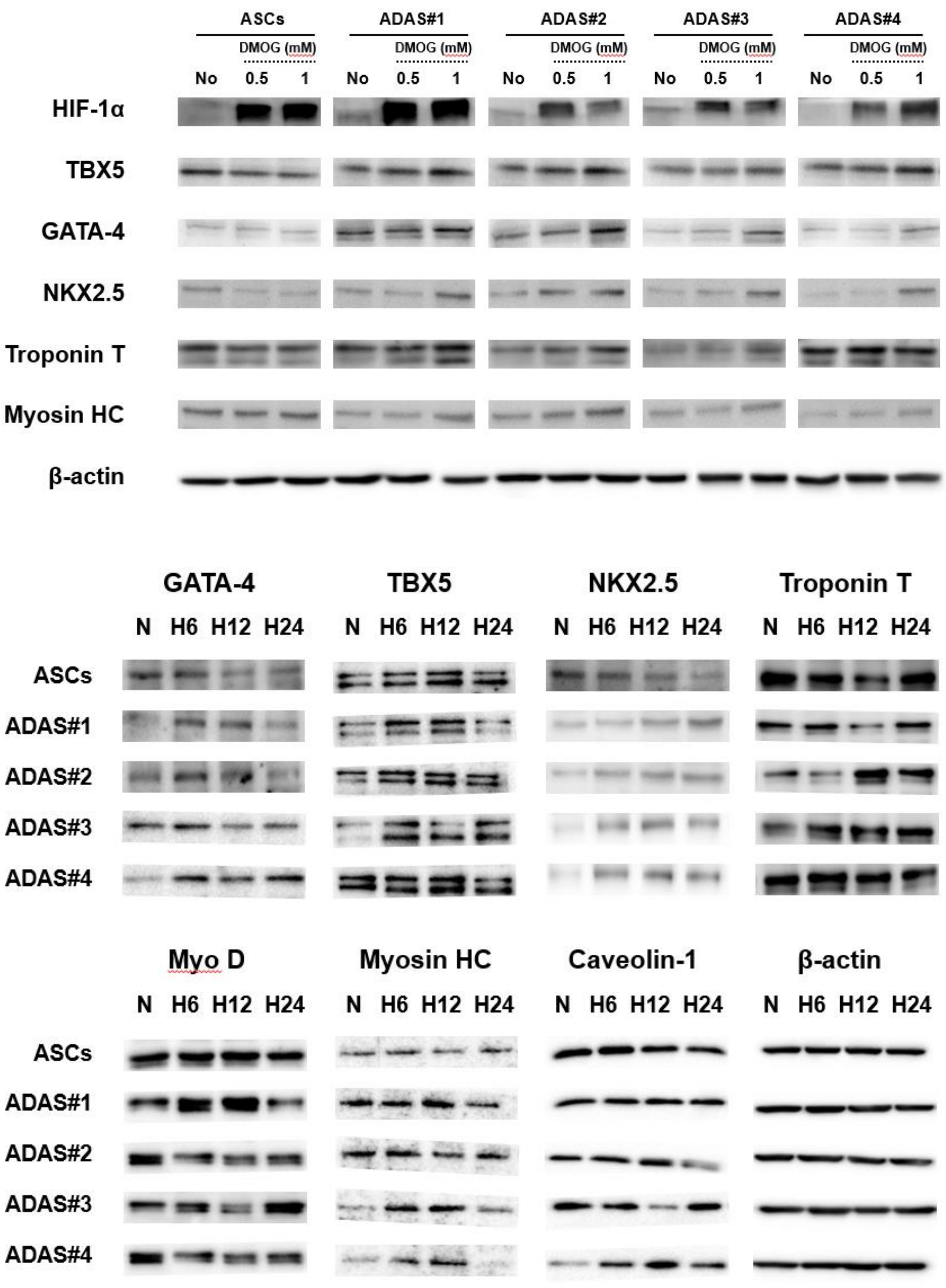

Figure S1. Western blot images of Figure 3 and Figure 5B.
